# Cathode Design Optimization toward the Wide-Pressure-Range Miniature Discharge Ion Source for a Vacuum Micropump

**DOI:** 10.3390/s19030624

**Published:** 2019-02-01

**Authors:** Tongtong Yao, Fei Tang, Jian Zhang, Xiaohao Wang

**Affiliations:** State Key Laboratory of Precision Measurement Technology and Instruments, Department of Precision Instrument, Tsinghua University, Beijing 100084, China; yaott16@mails.tsinghua.edu.cn (T.Y.); zhj4545@126.com (J.Z.); xhwang@mail.tsinghua.edu.cn (X.W.)

**Keywords:** vacuum MEMS, ion source, discharge, atmosphere to high vacuum, vacuum micropump

## Abstract

It is difficult to generate and maintain the vacuum level in vacuum MEMS (Micro-Electro-Mechanical Systems) devices. Currently, there is still no single method or device capable of generating and maintaining the desired vacuum level in a vacuum device for a long time. This paper proposed a new wide-pressure-range miniature ion source, which can be applied to a vacuum micropump. The miniature ion source consists only of silicon electrodes and a glass substrate. Its operating pressure range covers seven orders of magnitude, starting from atmospheric pressure, a promising solution to the difficulty. Based on the principle of gas discharge, the ion source features a simple two-electrode structure with a two-stage electrode spacing, operating under DC voltage excitation. The first-stage electrode spacing of the ion source is small enough to ensure that it starts working at atmospheric pressure down to a certain reduced pressure when it automatically switches to discharge at the larger second-stage electrode spacing and operates from that pressure down to a high vacuum. Two configurations of the ion source have been tested: without-magnet, operating from atmospheric pressure down to 1 mbar; and with-magnet, operating from atmospheric pressure to 10^−4^ mbar, which covers seven orders of magnitude of pressure. The ion source can be applied not only to a MEMS ion pump to meet demands of a variety of vacuum MEMS devices, but can also be applied to other devices, such as vacuum microgauges and mass spectrometers.

## 1. Introduction

Since their reliability and performance are greatly influenced by the ambient pressure and components, MEMS (Micro-Electro-Mechanical Systems) vacuum devices need to work under a certain vacuum level. Numerous MEMS devices, such as gyroscopes [1], optical switches [2] and accelerometers [3], require pressure at a level of about 10^−3^ to 2 mbar. Many other MEMS devices, such as pressure sensors [4], bolometers [5], atomic clocks [6,7], field emission sources and displays [8,9] and X-ray tubes [10], require a higher vacuum level. In addition, some significant MEMS analytical instruments require continuous air pumping (such as gas chromatographs [11,12]) or a high-vacuum environment (such as MEMS mass spectrometers [13,14,15] and MEMS transmission electron microscope [16]). The most widely accepted methods used to generate a vacuum in MEMS devices are vacuum encapsulation methods [17,18] and MEMS getter techniques [19,20]. However, the lowest pressure they can reach is about 10^−3^ mbar and the pressure cannot be regulated after sealing. Besides, it is difficult to maintain the vacuum level for a long time mainly due to gassing from the inner surface, outgassing of materials and significant gas permeation from the outside environment [7,21]. Therefore, the most promising solution to these issues is to pump air continuously via a vacuum micropump to generate and maintain the vacuum degree desired in a MEMS device.

The main types of vacuum micropumps include micro diaphragm pumps [22,23], micro vapour-jet pumps [24,25], micro cryopumps [26], Knudsen pumps [27,28,29] and micro ion pumps [30,31,32,33,34]. Micro diaphragm pumps, micro vapour-jet pumps and micro cryopumps can start working at atmospheric pressure and pump gas continuously. However, their final pressure is usually higher than 200 mbar, and it is difficult for this to be reduced further [34]. Knudsen pumps can also start working at atmospheric pressure but their final pressure is lower. A 162-stage Knudsen pump [27] achieved by S. An et al. has a final pressure of 1.2 mbar. Unfortunately, limited by temperatures and materials, it is difficult for Knudsen pumps to reach a lower pressure. At present, a high vacuum can be realised only by ion micropumps. It is worth noting that, ion pumps need to start at a low pressure provided by backing pumps, which greatly limits their use. T. Grzebyk et al. presented a high-vacuum MEMS ion-sorption pump [32] which needs to start at a pressure lower than 2 mbar. Currently, the only way to pump gas from atmospheric pressure to a high vacuum is to connect two different ion micropumps in series [35]. However, this method needs a very complicated fabrication process and is cumbersome in operation and requires manual switching between the two pumps during pressure reduction. It is worth noting that the main reason why ion micropumps are difficult to operate from atmospheric pressure to a high vacuum is that it is difficult to ionize gas particles over such a wide pressure range when using an ion source of the same structure. Therefore, to achieve a single high-vacuum ion pump starting from atmospheric pressure, it is necessary first to design an ion source that can operate in the pressure range from atmospheric pressure to a high vacuum.

There have been many studies on the ionization of gas particles under high pressure to atmospheric pressure, but there are fewer ion sources that can operate over a wide pressure range [36]. N. Miura et al. used a 900 MHz microstrip split-ring resonator to excite the microplasma [37], which operated between 133 and 10^3^ mbar. J. Cech et al. presented a wide-pressure-range plasma source based on a coplanar dielectric barrier discharge [38], which could operate between 0.1 and 10^3^ mbar when excited by a high voltage with a frequency of 40 kHz and 40 kV peak-to-peak value. T. Grzebyk et al. proposed a magnetron-like miniature ion source [39]; its three-electrode configuration with two symmetrical magnets could operate in the pressure range from 10^−5^ to 70 mbar excited by a DC voltage. Currently, there is no ion source that can operate from atmospheric pressure to high vacuum. However, the work in Reference [39] provides a good reference.

This paper proposes a two-electrode structural MEMS ion source based on gas discharge, which can operate over a wide pressure range from atmospheric pressure to high vacuum. The principal analysis and simulation of the ion source have been carried out. The ion source has been fabricated and two configurations (with- and without-magnet) have been tested. The influences of electrode spacing and magnetic field on the operating characteristics of ion sources have been analyzed. The next step is to take the ion source as the crucial component of the ion micropump, which allows the micropump to operate from atmospheric pressure to a high vacuum, to satisfy the demands of various MEMS vacuum devices.

## 2. Device Construction and Working Principle

**Figure 1:**
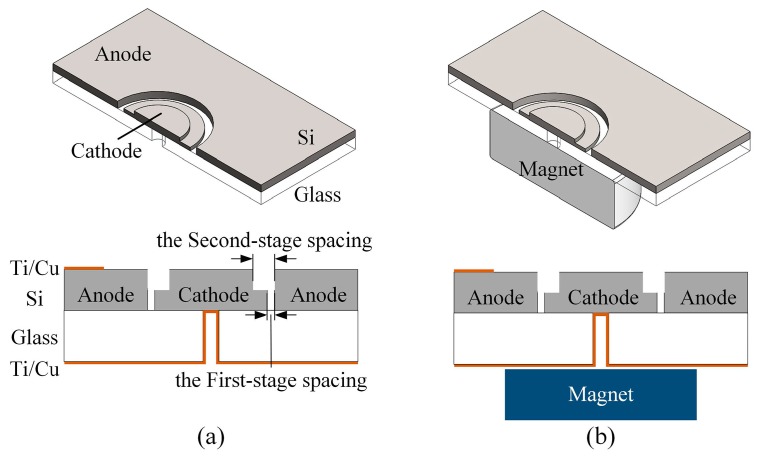
The schematic diagram of ion source structure: (**a**) without-magnet configuration; (**b**) with-magnet configuration.

The ion source consists of silicon electrodes and a glass substrate and was fabricated using MEMS technology. The electrodes feature a two-electrode structure, with a central cathode and a surrounding anode, as shown in Figure 1. The anode features a cylinder shape at its inner side and the cathode is a two-stage stepped cylindrical structure, which successfully gives the ion source a two-stage electrode spacing. The spacing area of each stage is in a ring shape to ensure uniform discharge. The first-stage and second-stage electrode spacings of the ion source are 40 µm and 460 µm, respectively; the radii of the two-stage stepped cathode are 1.94 mm and 1.52 mm. The silicon electrodes are bonded to the glass substrate and the overall size of the ion source is 12 × 11 × 0.8 mm^3^. Two configurations of the miniature ion source have been investigated: with- and without-magnet configurations. The only difference between the two configurations is that a neodymium-iron-boron magnet is placed directly under the structure in the with-magnet configuration, generating a magnetic field of about 0.3 T orthogonal to the glass-silicon interface.

**Figure 2:**
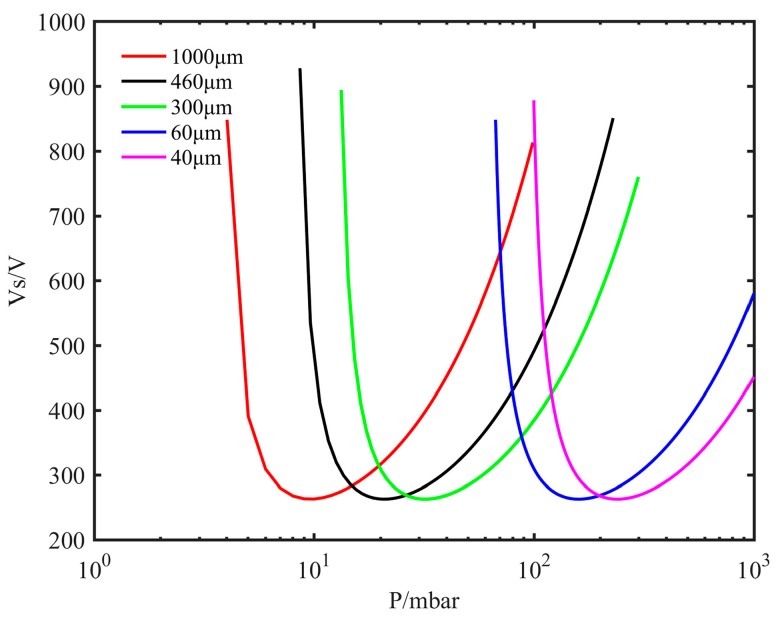
The Paschen curves for different distances.

The miniature ion source is designed based on the principle of gas discharge [40]; according to the Paschen law, the expression of gas breakdown potential is shown as follows:

(1) $$V_{s}=\frac{CPd}{\ln(APd)-\ln\left[\ln(1+1/\gamma )\right]},$$

where *P* is the pressure, *d* is the electrode spacing, and *A*, *C* and *γ* are constants related to the properties of gas and electrode. Therefore, the breakdown voltage is a function of *Pd* only, i.e. a function of the pressure multiplied by the electrode spacing. Discharge is apt to occur between electrodes within a certain *Pd* range. Therefore, when the electrode spacing *d* is different, the pressure range *P* in which the discharge is apt to occur is different. If *d* is small, the value of *P* at which the discharge is apt to occur is mainly in the high-pressure range; if *d* is relatively large, discharge is easier in a low-pressure range. After calculation, Paschen curves for various distances are shown in Figure 2. The ion source can operate over a wide pressure range starting from atmospheric pressure by setting values of electrode spacing reasonably. Therefore, we designed the two-stage electrode spacing of the structure. For the first-stage spacing (40 µm), the pressure range for easy discharge is from atmospheric pressure to about 100 mbar; for the second-stage spacing (460 µm), the pressure range for easy discharge is about 200–5 mbar. Therefore, the two-stage electrode structure can discharge in a wide range of pressure values from atmospheric pressure down to about 5 mbar.

The ion source ionizes gas particles by electrical discharge. By applying a DC voltage between two electrodes, primary electrons obtain sufficient energy to collide with gas particles and each collision will generate ions and additional electrons. Meanwhile, secondary electrons are generated after the ions bombard the cathode surface. These two processes lead to a self-sustaining process of discharge.

**Figure 3:**
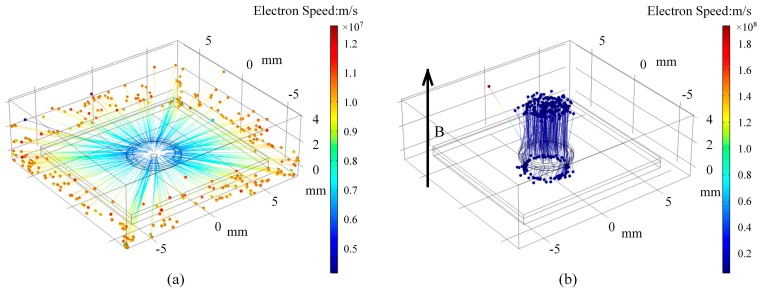
The simulation results of electron motion trajectory: (**a**) without a magnetic field; (**b**) with a magnetic field of B = 0.3 T.

Compared to the without-magnet ion source, the with-magnet ion source has exactly the same structure except that a neodymium-iron-boron magnet is put directly under the structure so that electrons are affected by electric and magnetic fields. Forced by the magnetic field, electrons execute a spiral motion due to the Lorentz force, with its motion radius:

(2) $$r=\frac{1}{B}\sqrt{\frac{2mV}{q}},$$

where *m* is the electron mass, *q* is the electron charge, *B* is the magnetic flux density, and *V* is the potential difference between the cathode and anode. For *V* = 900 V and *B* = 0.3 T, *r* is equal to 340 µm, which is far smaller than the diameter of the anode. A 3D model of the miniature ion source was built in the COMSOL Multiphysics software to simulate the motion trajectory of the electrons. The voltage between the cathode and anode was set to 900 V and the trajectories of the electrons were obtained without magnetic field and with a magnetic field of about 0.3 T, as shown in Figure 3. When there is no magnetic field, the electrons rush directly to the periphery, beyond the boundary of the ion source (Figure 3a). Figure 3b shows that the presence of the magnetic field changes the direction of motion of the electrons, limiting the electron motion to the space above the cathode. It is obvious that in the effective operating area, the electron trajectories of the with-magnetic configuration are longer than that of the without-magnetic configuration. Moreover, it can be seen from Equation (2) that the magnetic field makes the electrons spiral, making the trajectories of the with-magnetic configuration much longer. Unfortunately, due to the limitations of the simulation steps, we cannot see the complete trajectory of the electron spiral motion. Therefore, in the presence of a magnetic field, the probability of collisions of electrons with gas particles will be increased greatly and the discharge can still occur and be maintained even under the condition of a much lower pressure, so the operating pressure of the ion source can be further reduced and can still operate under high-vacuum conditions.

## 3. Fabrication

**Figure 4:**
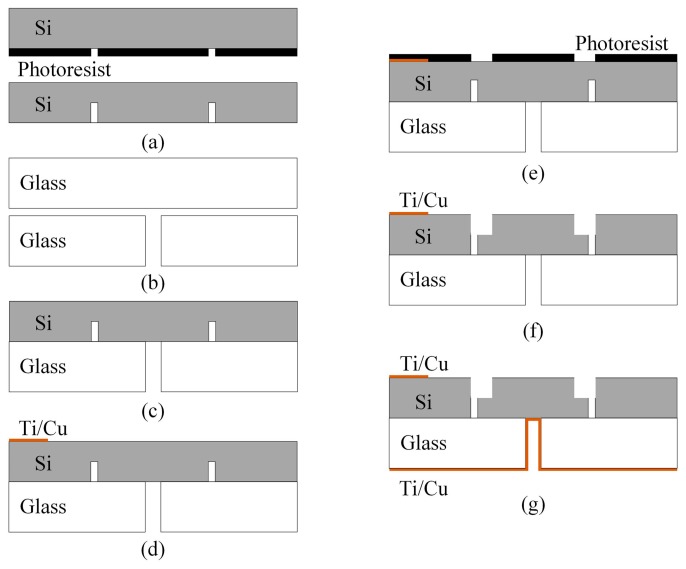
The main steps of fabrication of the miniature ion source.

The ion source was fabricated by using MEMS technology. A 4 inch (100)-oriented, 300 μm thick, low resistivity (0.002–0.004 Ω·cm), double-side-polished silicon wafer and a 4 inch, 500 μm thick Borofloat 3.3 glass were used. First, the first-stage electrode spacing was obtained by ICP (inductively coupled plasma) etching, with a 150 µm etching depth, after masking by a positive photoresist and photolithographically patterned (Figure 4a). Then a conical through-hole is fabricated on the glass substrate by laser processing (Figure 4b). This was followed by the anodic bonding of the silicon layer (the photoresist had been removed) with the glass substrate (800 V, 4 mA, 340 °C, 10 min, Figure 4c). Next, a Ti/Cu (300 Å/5000 Å) contact to the anode electrode was formed on the top side of the structure by using a lift-off process (Figure 4d). Then, a positive photoresist was used as a mask and photolithography was used to obtain the desired pattern (Figure 4e). The second-stage electrode spacing was then obtained after ICP etching, with a 150 µm etching depth (Figure 4f), so obtaining the completely separated anode and cathode. Lastly, a Ti/Cu (300 Å/5000 Å) layer was sputtered onto the bottom of the glass substrate, the through-hole allowing the bottom Ti/Cu layer to be in direct contact with the cathode (Figure 4g).

## 4. Results and Discussion

The miniature ion source structures were placed in a reference vacuum chamber with a precisely controlled pressure for testing. The vacuum chamber is a cuboid cavity with an internal dimension of 145 × 150 × 80 mm^3^. The circumference and bottom surfaces of the cavity are integral and made of stainless steel. After an ion source structure was placed in the chamber, tempered glass was placed over the cuboid cavity and the chamber was sealed by using a Viton O-ring. The vacuum chamber is connected to a pumping system consisting of a molecular pump HiPace 10 (Pfeiffer Vacuum GmbH, Wetzlar, Germany) and a diaphragm pump MVP006-4 (Pfeiffer Vacuum GmbH) in series. The pressure in the chamber is controlled by a Granville-Phillips 203 variable leak valve (MKS Instruments, Inc., Andover, MA, USA). One end of the valve is connected to the chamber and the other end is directly exposed to the air. The pressure in the chamber is measured by the vacuum gauge PKR361 (Pfeiffer Vacuum GmbH) and read out by the controller TC110 of the HiPace 10.

The cathode of the ion source was grounded, the anode was biased positively by a DC voltage U supplied by a high DC voltage source (Dong Wen High Voltage (Tianjin) Co., Ltd., Tianjin, China), and a 1 MΩ resistor was connected in series in the circuit to measure and limit the discharge current generated from ionization during the operation of the ion source. A Fluke 17B digital multimeter (Fluke Co., Avery, WA, USA) was used to measure the voltage across the resistor and the voltage was divided by 1 M to get the discharge current value. What is more, the tempered glass above the vacuum chamber serves as a window for observing and capturing the discharge images, about 70 mm away from the surface of the ion source, and the image is taken with a built-in camera of an iPhone 6s (Apple Inc., Cupertino, CA, USA).

### 4.1. Test of without-Magnet Configuration

**Figure 5:**
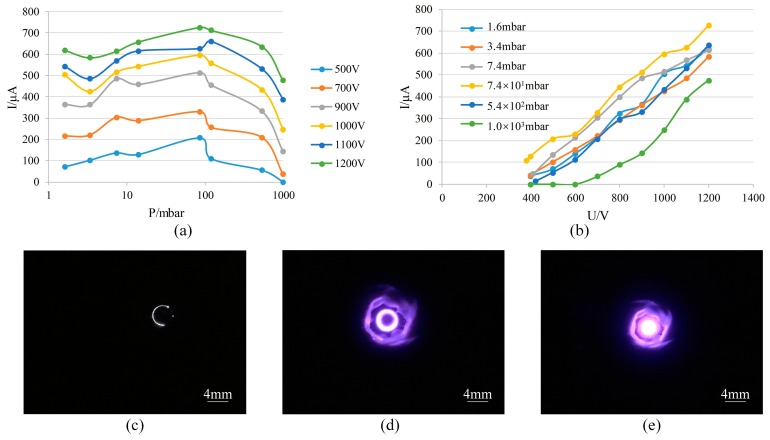
The measurement of the properties of the without-magnet ion source (**a**,**b**) and discharge pictures at different conditions (**c**–**e**): (**a**) discharge current vs. pressure; (**b**) discharge current vs. voltage U; (**c**) p = 1 atm, U = 800 V; (**d**) p = 14 mbar, U = 700 V; (**e**) p = 7.4 mbar, U = 700 V.

**Figure 6:**
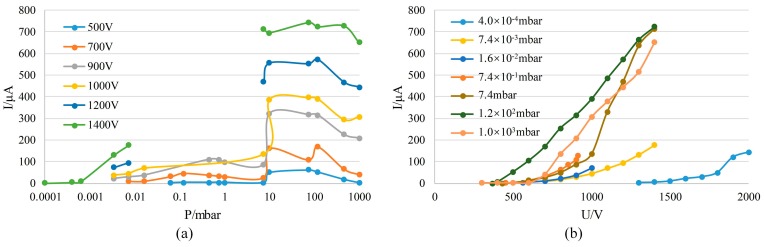
The measurement of the properties of the with-magnet ion source: (**a**) the relation between discharge current and pressure; (**b**) the relation between discharge current and voltage U.

The discharge current was measured as a function of pressure at different constant voltages U (Figure 5a. Each discharge current value in Figure 5, Figure 6, Figure 9 and Figure 10 is the average of the five data obtained during tests.) and showed that the minimum operating pressure of the ion source was down to 1 mbar. Operation of the test structure was started at atmospheric pressure and the gas discharge was observed, which occurred mainly in the space in the first-stage electrode spacing (Figure 5c). On reducing the pressure to about 100 mbar, the current reached a maximum value, at which point the discharge was automatically transferred to the space in the second-stage electrode spacing, and the discharge could still ignite as the pressure decreased (Figure 5d). When the pressure was lower than 10 mbar, discharge occurred in the space above the cathode (Figure 5e). A discharge current with the increasing voltage U was also measured (Figure 5b), which showed that the discharge intensity was enhanced and current rose as the voltage increased. However, when the pressure was lower than 1 mbar, discharge between the electrodes was unstable. With rising voltage, the discharge between electrodes disappeared and discharge occurred between the anode and the reference vacuum chamber wall. It is known from the Paschen curve that this is because the discharge is apt to occur between electrodes with a larger spacing when pressure is lower than 1 mbar. In our experimental setup, the reference vacuum chamber wall was grounded and the distance between the chamber wall and the anode was much greater than that between the anode and the cathode. Under this pressure condition, the reference vacuum chamber wall is equivalent to a cathode so that discharge occurred between it and the anode.

Therefore, the test result shows that the without-magnet ion source can start operating from atmospheric pressure and tends to operate in low to medium vacuum.

### 4.2. Test of with-Magnet Configuration

**Figure 7:**
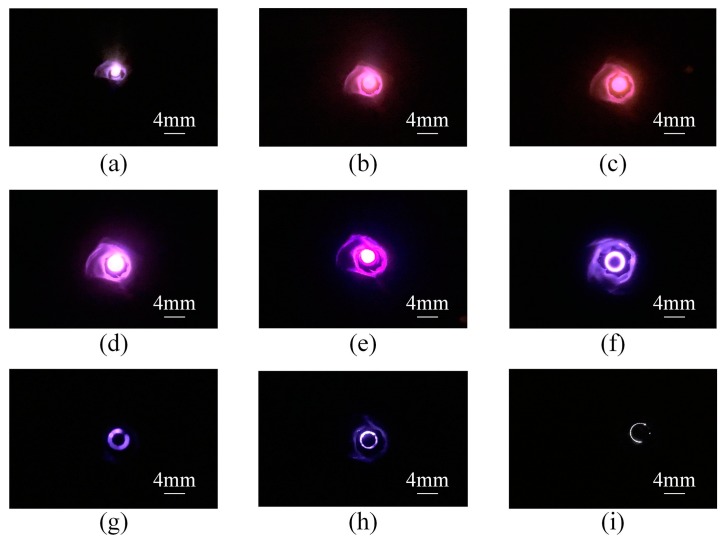
The images of ion source discharge at different pressures (mbar): (**a**) 6.3 × 10^−4^; (**b**) 7.4 × 10^−3^; (**c**) 1.6 × 10^−2^; (**d**) 4.6 × 10^−1^; (**e**) 7.4; (**f** ) 1.4 × 10^1^; (**g**) 1.2 × 10^2^; (**h**) 5.4 × 10^2^; (**i**) 1 × 10^3^.

Then, a neodymium-iron-boron magnet (0.3 T) was placed at the bottom of the ion source structure for testing. The discharge current was measured as a function of pressure at different constant voltages U (Figure 6a). It showed that the with-magnet ion source started operating at atmospheric pressure. When the pressure was about 100 mbar, the discharge current reached a maximum value; after the pressure was lower than 10 mbar, discharge switched automatically from the space between the electrodes to the space above the cathode, and featured a weakened discharge intensity and decreased current. When the voltage U was kept at 1400 V, the glow discharge was observed at pressure even as low as 10^−4^ mbar, and the discharge current was still up to around 5 µA when the pressure was 4 × 10^−4^ mbar. The reason behind this is that the motion of electrons is affected by electric and magnetic fields when a magnet is present. The presence of a magnetic field allows ion sources to operate well in a high vacuum, which is consistent with the theoretical analysis and simulation results. We also measured the relationship between the discharge current and voltage U under different pressures as the voltage increased (Figure 6b). In addition, the discharge appearance when the ion source was operating under different pressure conditions was recorded (Figure 7).

Figure 6a shows that discharge currents between electrodes are not obtained in the 0.02–10 mbar pressure range when the voltage was 1200 V or 1400 V. This is because discharges occurred between the anode and the reference vacuum chamber wall in these cases. The reason for this phenomenon is similar to the case where the without-magnet configuration could not discharge normally under a pressure of lower than 1 mbar. In this pressure range, discharge is apt to occur between electrodes with a larger spacing. Although the presence of a magnet can restrict the range of motion of electrons, the intensity of discharge is much higher and a magnetic field of 0.3 T is unable to trap electrons when the voltage exceeds 1000 V. However, if the magnetic flux density increases, the restraining force for electrons will be enhanced, so that the above phenomenon can be avoided, and the discharge can occur between electrodes; that is, the ion source can operate as expected.

Therefore, the test result shows that the with-magnet ion source can operate over a wide pressure range from atmospheric pressure to high vacuum, covering seven orders of magnitude of pressure.

### 4.3. Test of the Ion Source Stability

**Figure 8:**
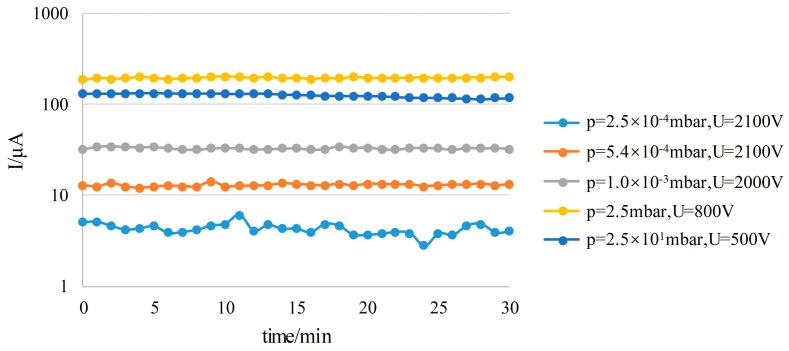
The stability of the ion source operation.

Moreover, the discharge currents obtained are very stable over time. A with-magnet ion source structure (the first-stage and second-stage electrode spacings are 60 µm and 340 µm, respectively) was tested. The discharge current was monitored for 30 minutes at 5 different levels of pressure. The monitoring results show that the fluctuations of the current values at different pressures are very small (Figure 8), and the tests showed no degradation of the ion source for at least 30 min of continuous operation.

### 4.4. Influence of Electrode Spacing

**Figure 9:**
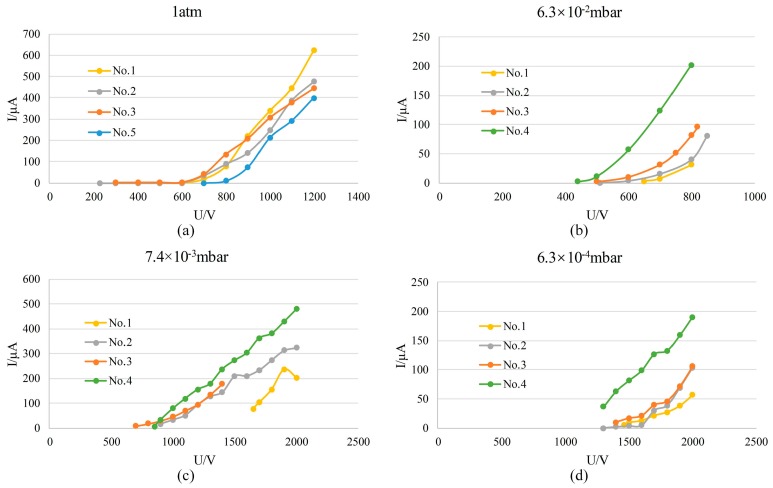
The discharge currents of different devices vs. voltage.

Several ion sources with different electrode spacing dimensions were designed, to test the influence of different sizes on the discharge intensity of the ion source under different pressures. The two-stage electrode spacing of the ion source was designed in the following combinations: 40/340 µm (No. 1), 40/400 µm (No. 2), 40/460 µm (No. 3), 40/500 µm (No. 4), and 60/460 µm (No. 5). According to the test results reported in Section 4.1 and Section 4.2, it is known that only the first-stage electrode spacing is responsible for discharge from atmospheric pressure down to about 100 mbar. We can learn more from the test results of different devices that the ion sources with 40 µm or 60 µm first-stage spacing are all able to discharge under atmospheric pressure, and that the discharge intensity of ion sources Nos. 1, 2 and 3 are stronger than that of No. 5 (Figure 9a), i.e., the ion source with a 40 µm electrode spacing is more apt to discharge than that with a 60 µm spacing. When pressure is lower than 6.3 × 10^−2^ mbar, discharge currents of ion sources Nos. 4, 3, 2 and 1 decrease successively under different pressures, and the discharge intensity weakens (Figure 9b–d); i.e., the discharge intensities of the ion sources with 500, 460, 400 and 340 µm electrode spacings weaken successively when pressure is lower than 6.3 × 10^−2^ mbar, indicating that the greater the electrode spacing, the easier the discharge in the low-pressure range. In the middle range of the pressure (about 100 mbar–6.3 × 10^−2^ mbar), the device selects the mode of discharge that is the easiest based on the different cooperation between the two-stage spacing, and no obvious rules are observed. We can see that the size of the electrode spacing has a great influence on the operating performance of the ion source. In order to operate over a wider range of gas pressures, the ion source preferably has a small first-stage electrode spacing and a relatively large second-stage electrode spacing. The two-electrode setup in Reference [39] only has one stage electrode spacing and the operating pressure range is much smaller than the ion source herein. Additionally, compared with the shape of electrode spacing close to a square in Reference [39], we believe that designing the electrode spacing as a ring makes the discharge more stable and also improves the performance of the ion source.

### 4.5. Influence of Magnetic Field

**Figure 10:**
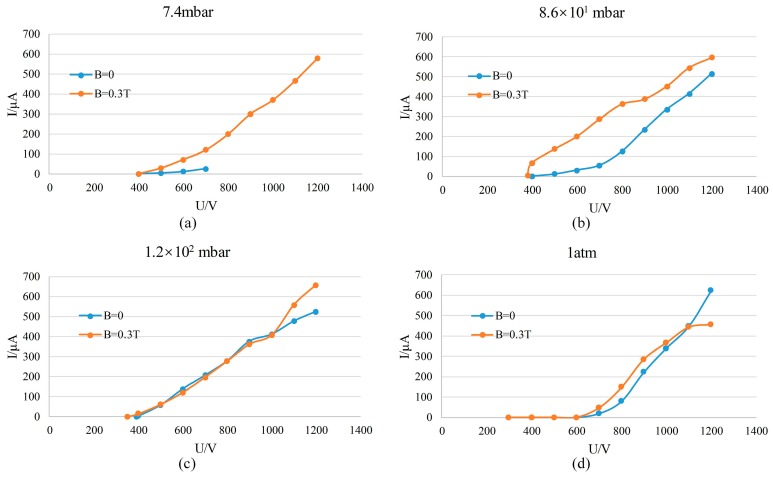
The discharge currents of with- and without-magnet configurations vs. voltage: (**a**) p = 7.4 mbar; (**b**) p = 8.6 × 10^1^ mbar; (**c**) p = 1.2 × 10^2^ mbar; (**d**) p = 1 atm.

Comparing the test results of with- and without-magnet configuration, it is obvious that the existence of a magnetic field greatly decreases the minimum operating pressure of the ion source, widening the range of pressure at which the ion source operates. In order to analyze further the influence of the magnetic field on the operation of the ion source, more tests were carried out. Two configurations (with- and without-magnet) of the ion source No. 1 (40/340 µm) were tested. According to the test results, discharge currents of the with-magnet configuration are obviously higher than those of the without-magnet configuration when pressure is lower than 100 mbar (Figure 10a,b). The reason behind this is that the presence of a magnet increases the probability of ionization, thereby increasing the discharge current. When the pressure is higher than 100 mbar, the discharge currents of the two configurations differ slightly (Figure 10c,d). This is because in high-pressure environments, the number of gas particles is very large, so there will be many collisions during the motion of the electrons. The electron trajectory is affected mainly by the collisions, so the magnet has little effect on the discharge current. According to the above analysis, if a magnet with a larger magnetic flux density is used, the minimum operating pressure of the ion source could be reduced further, and discharge currents of the ion source in a low-pressure environment could be increased. It should be noted that the comparison between the results of Figure 6a and Figure 5a seems to be inconsistent with the results of Figure 10. This is because Section 4.1 and Section 4.2 use different devices (although they are theoretically the same size, they are different because they are laboratory-made devices), while the tests in Figure 10 use the same device. Therefore, it is credible to use the results of Figure 10 to illustrate the influence of magnetic fields.

## 5. Conclusions

In this paper, a wide-pressure-range miniature ion source is proposed, which is fabricated by MEMS technology and consists of a glass substrate and silicon electrodes. The electrodes of the miniature ion source feature a two-electrode structure, and the electrode spacing is of a circular ring shape, which ensures the uniformity of discharge during operation of the ion source. The ion source has a two-stage electrode spacing. The first-stage electrode spacing is small enough to ensure that the ion source starts operating at atmospheric pressure. Under high pressure, the discharge occurs mainly in the first-stage spacing area. Under a lower pressure, discharge switches automatically to the second-stage spacing area. Two different configurations (with- and without-magnet) of the ion source were tested. The test result shows that the minimum operating pressure of the without-magnet configuration can be down up to 1 mbar; the with-magnet configuration has a minimum operating pressure of 10^−4^ mbar, with its operating pressure range covering seven orders of magnitude. Besides, the presence of a magnet is able to increase the discharge currents of the ion source. By not using fragile cathode materials, the ion source can operate stably for a long time. It is promising to realize a high-vacuum pump starting from atmospheric pressure to ensure the generation and maintenance of high vacuum in a MEMS device. In addition, the ion source is technically compatible with other MEMS devices, so it can also be applied to miniature devices such as micro vacuum gauges, micro mass spectrometers and plasma display panels.
